# Food cultures, lifestyles and health of Pacific adolescents: Questionnaire data and anthropometric measurements focused on 11 to 16 years old students living in New Caledonia

**DOI:** 10.1016/j.dib.2025.112299

**Published:** 2025-11-20

**Authors:** Guillaume Wattelez, Stéphane Frayon, Akila Nedjar-Guerre, Corinne Caillaud, Olivier Galy

**Affiliations:** a University of New Caledonia, Noumea, New Caledonia; b Interdisciplinary Laboratory for Research in Education, University of New Caledonia, Noumea, EA7483, New Caledonia; c Sydney School of Public Health, Faculty of Medicine and Health, The University of Sydney, New South Wales, Australia; d Charles Perkins Centre, The University of Sydney, New South Wales, Australia

**Keywords:** Pacific island countries and territories, Melanesia, Health, Behavior, Nutrition, Impedancemeter measurements, media exposure, teenagers

## Abstract

This paper presents data collected from 1062 adolescents (521 females, 540 males) attending eight different schools in New Caledonia, an archipelago of the South Pacific. The adolescents, aged from 11 to 16 years old, lived in rural (*n* = 384) or urban (*n* = 678) areas of this French territory. The data, collected from July 2018 to April 2019, includes information from several different sources.

Demographic information (sex, age and socioeconomic status) was obtained from the school management system. Anthropometric measurements (height, weight, waist size, skinfold thickness and impedancemeter measurements) were obtained and then derived to assess weight status components (z-score and weight status categories) following standard references. Bioimpedance and skinfold thickness were also used to assess body composition.

The questionnaires were designed to collect important information from participants about their lifestyles with the capacity to relate the information to cultural identity. Participants were asked to self-identify to an ethnic community. Participants eating behavior was assessed via the Food Frequency Questionnaire (FFQ). The self-esteem was assessed using a validated French translation of the well-known Rosenberg's self-esteem scale. The prevalence and underlying reasons for procrastination among students was evaluated using the Procrastination Assessment Scale for Students (PASS). The sleep questionnaire was built to calculate sleep time in participants and identify underlaying reasons for insufficient sleep. The digital device and media questionnaire primarily aimed to investigate digital devices’ access and ownership (Multimedia devices), screen time (Screens and me), main purpose of use and the influence of digital and non-digital media on food choices (Screen and information). The “My body and me” questionnaire is a validated questionnaire that determines participant’s physical self-perception profile. Data about internalization of societal standards of appearance and the perceived pressures they experience to conform to these standards were collected using the Sociocultural Attitudes Towards Appearance Questionnaire-4 (SATAQ-4). Finally, the self-perceived appearance questionnaire aimed to assess the discrepancy between the participants’ self-perceived and actual weight status.

Finally, information about aerobic fitness (i.e., maximal aerobic speed test and assessment of maximal oxygen consumption) is known for 334 participants.

The dataset contains sensitive data that are provided through restricted access. Additionally, a subset of non-sensitive data can be accessed openly.

The paper describes the file’s content and the characteristics of the participants. This dataset contains diverse high-quality data valuable for researchers, public authorities and other parties interested in behavior, lifestyles and health of Pacific adolescents.

Specifications Table

**Table:** 

Subject	Health Sciences, Medical Sciences & Pharmacology
Specific subject area	Behaviors, habits, lifestyle and health of adolescents living in the Pacific, especially in New Caledonia
Type of data	Raw, Processed (.xlsx and .csv files).
Data collection	Participants were recruited in 8 secondary public schools of New Caledonia randomly selected according to their location (rural/urban and Province), size (*n* > 200) and following the school staff agreement [1]. All carers or parents as well as the participants provided a written consent before participating in the study. The measurements and questionnaires were conducted during class time in the presence of a research team member. The anthropometric measurements were taken by or with the school nurse while the participants wore light clothes. Leicester Tanita HR 001 was used for height measures, Tanita
	DC-430MA was used for impedancemeter measures and Holtain skinfold callipers were used for skinfold thickness.
Data source location	Country: New Caledonia Towns/Villages: Dumbéa, Koumac, Lifou, Nouméa, Païta, Poindimié
Data accessibility	Repository name: Zenodo Data identification number: Restricted data: 10.5281/zenodo.13413392 Open data: 10.5281/zenodo.15485833 Direct URL to data: **R**estricted data: https://doi.org/10.5281/zenodo.13413392 Open data: https://doi.org/10.5281/zenodo.15485833 Instructions for accessing these data: An embargo has been applied on the public data that will be freely and openly available under the CC-BY 4.0 licence on 30 June 2026. The public data is an anonymized version of the restricted data. Restricted data can be accessed on demand with the Zenodo user system management depending on the seriousness of the request. The generic Data Use Agreement is in supplementary materials. However, specific conditions of access may be specified according to the studies and processes envisaged. The minimum requirement in terms of ethics is General Data Protection Regulation (GDPR) compliance and no re-identification process. The team providing the restricted data also expects collaboration or acknowledgment (to be discussed with the demanders).
Related research article	S. Frayon, G. Wattelez, E. Paufique, A. Nedjar-Guerre, C. Serra-Mallol, O. Galy, Overweight in the pluri-ethnic adolescent population of New Caledonia: Dietary patterns, sleep duration and screen time, The Lancet Regional Health - Western Pacific 2 (2020) 100,025. https://doi.org/10.1016/j.lanwpc.2020.100025. [1]

## 1 Value of the Data

• There is no other data set providing information about health-related behaviors, food intake and weight status in a large group (1000 participants) of New Caledonian adolescents living in diverse contexts (urban, remote or tribal environments).
• The comprehensive set of questionnaires used to collect data also provides an overview of some psychological characteristics of the participants. With the rise in poor mental health in young people, it can fill a gap for researchers interested in this area. Researchers in many scientific fields, such as nutrition, psychology, health perception, computer science, etc. may find in this dataset unique information from an under-studied population.
• The data may be used by public health and computer science researchers to test new hypotheses or develop models for lifestyle interventions related to mental health or health-related behaviors. For instance, researchers could explore associations between the use of screens (including screen time and the actions taken with digital devices) with procrastination or self-esteem.
• This data is a valuable resource for researchers, public authorities and stakeholders interested in public health since it may be used for comparison with other location and it could be of historical interest, or at least of archival interest for comparison with a future situation, especially as these data were collected shortly before the COVID-19 pandemic.

## 2 Background

In several Pacific countries, >50 % of the total population and 30 % of the adolescent population is overweight or obese [1,2]. Some studies identified industrialization, westernization and globalization as key factors to this situation because they induced shifts in diet, food production and other habits [3].

New Caledonia is a South Pacific archipelago with a multicultural population also affected by the obesity pandemic. Practices differ greatly depending on the place of living (rural/urban) and the cultural background. The aims of this dataset are (1) to inform on the eating habits of the adolescents from diverse cultural background and place of living, (2) to associate these factors with their weight status and (3) to describe adolescents’ attitudes to food, their weight and their use of social networks to identify educational tools for tackling these complex issues.

The data collection was based on both objective measurements with scientific devices for the anthropometric data which are derived in particular to obtain weight status and subjective data with questionnaires validated by many research fields and translated in French.

## 3 Data Description

### 3.1 Repository and file description

Table 1 describes the repositories that make up the dataset [4,5]. The restricted non-anonymized repository contains the full dataset and the open anonymized repository contains only a selection of non-identifying information. As it is generally accepted particularly in the context of the General Data Protection Regulation (GDPR), we consider in this paper that a dataset is anonymized when the reidentification risk is very low, even by using additional data from external sources. On the contrary, a dataset is considered non-anonymized when the risk of identification is not negligible, bearing in mind that additional information from other sources could be used to identify individuals.

**Table 1:** List of the repositories included in the dataset, their content and DOI.

Repository title	Content	DOI
Anthropometric measurements, questionnaire responses and fitness data collected during the scientific project: "Eating cultures and behaviors of young people in French-speaking Pacific countries in the 21st century: the example of New Caledonia" (non-anonymized version) [4]	Full dataset with information on participants, anthropometric measurement and answers to questionnaires. Six files are in the repository: • two files with primary answers in French (xlsx and csv format) • two files translated in English (xlsx and csv format) • two files with coded answers: label text values in the questionnaire responses have been replaced by numbers and dummy variable columns have been added in case of multiple selection (xlsx and csv format)	10.5281/zenodo.13413392
Anthropometric measurements, questionnaire responses and fitness data collected during the scientific project: "Eating cultures and behaviors of young people in French-speaking Pacific countries in the 21st century: the example of New Caledonia" (anonymized version) [5]	Part of the dataset with only information that cannot be used to identify the participants. Ten files are in the repository: • two files with primary answers in French (xlsx and csv format) • two files translated in English (xlsx and csv format) • two files with coded answers: label text values in the questionnaire responses have been replaced by numbers and dummy variable columns have been added in case of multiple selection (xlsx and csv format) • a data description file: full description of the columns found in the datafiles (pdf format) • two codebook files (an English and a French version) describing the questions and the response options (pdf format) • a data content file: brief description of the sample and graphs showing the distributions of selected items of the questionnaire (pdf format)	10.5281/zenodo.15485833

Table 2 describes the content of the data files and shows the restricted, partially open and open information. Some numeric data are partially open in the open dataset, meaning that an open access is offered for only 90 % of the female participants and 90 % of the male participants to avoid identification based on particular values.

**Table 2:** Description of the data file content and availability of the information.

Section	Content description	Type of data: categories/ranges	Availability
Participant description	Participant: Participant number		Open
	Place of living: Place of living according to school location	Category data: Rural; Urban	Restricted
	School: Visited school	Category data: Koumac; Koutio; Lifou; Magenta; Mariotti; Ste-Marie Païta; Poindimié; Tuband	Restricted
	Division: Class in which the participant was enrolled	Category data: depends on the school	Restricted
	Age range: Age range of the participant	Category data: Less than or equal to 12; Between 13 and 14 inclusive; Greater than or equal to 15	Open
	Age in years: Age in years of the participant determined by rounding off age in months divided by 12	Numeric data: [10,17]*	Restricted
	Age in months: Age in months of the participant	Numeric data: [126,199]	Restricted
	Sex: Sex of the participant	Category data: Female; Male	Open
	Legal representative 1 SES: Socioeconomic status of the first legal representative	Category data: A; B; C; D	Restricted
	Legal representative 2 SES: Socioeconomic status of the second legal representative	Category data: A; B; C; D	Restricted
	Retained SES: SES retained for the participant - the highest SES between SES of the legal representative 1 and the legal representative 2	Category data: A; B; C; D	Restricted
	International SES: International SES	Category data: High; Medium; Low	Restricted
	GeneActiv file: GENEActiv file (with no extension) associated with the participant The GENEActiv dataset is described in [6]	Text	Open
Anthropometric measurements	Height in m: Height of the participant in meters	Numeric data: [1.30,1.94]	Restricted
	Weight in kg: Weight of the participant in kilograms	Numeric data: [25.0131.6]	Restricted
	Waist size in cm: Waist size of the participant in centimeters	Numeric data: [53.2141.1]	Restricted
	Bicipital: Bicipital thickness in millimeters	Numeric data: [2.4,89.0]	Restricted
	Tricipital: Tricipital thickness in millimeters	Numeric data: [3.8,54.4]	Restricted
	Suprailiac: Suprailiac thickness in millimeters	Numeric data: [3.7,54.4]	Restricted
	Subscapular: Subscapular thickness in millimeters	Numeric data: [3.2,88.8]	Restricted
	Tanner stage: Tanner stage declared by the participant	Ordinal category data: integer from 1 to 5	Open
	Sum of skin folds in mm: Sum of skin folds (Bicipital + Tricipital + Suprailiac + Subscapular) in millimeters	Numeric data: [16.9243.6]	Partially open
	Density: Density of the participant assessed from the sum of skin folds and the sex	Numeric data: [0.99,1.08]	Partially open
	Fat body mass in %: Fat body mass (FBM) of the participant assessed by the formula using the density	Numeric data: [10.74,47.90]	Partially open
	Lean body mass in kg: Lean body mass (LBM) of the participant assessed by the formula using weight and the fat body mass (FBM) of the participant	Numeric data: [20.20,79.05]	Partially open
	Waist size-height ratio: Waist size/height (in cm)	Numeric data: [0.36,0.87]	Partially open
Impedancemeter measurements	MACHINE to CHECKSUM: Collection of variables provided by the TANITA scale records described in the instruction manual [TANITA user guide]	Category, numeric and date-time data	Open except MDATE, MTIME and AGE that are restricted and numeric data (HEIGHT, WEIGHT.kg, etc.) that are partially open
Weight status derived from anthropometric measurements	IOTF_L: IOTF Lambda index used to compute the IOTF z-score IOTF_M: Mu index used to compute the IOTF z-score IOTF_S: Sigma index used to compute the IOTF z-score IOTF_zScore: IOTF z-score IOTF_percentile: IOTF percentile based on IOTF z-score	Numeric data	Restricted
	IOTF Weight status: IOTF weight status based on IOTF percentile	Category data: Underweight; Normal; Overweight; Obese	Open
	Cachera_L: Cachera Lambda index used to compute the Cachera z-score Cachera_M: Cachera Mu index used to compute the Cachera z-score Cachera_S: Cachera Sigma index used to compute the Cachera z-score Cachera_zScore: Cachera z-score Cachera_percentile: Cachera percentile based on Cachera z-score	Numeric data	Restricted
	Cachera Weight status: Cachera weight status based on Cachera percentile	Category data: Underweight; Normal; Overweight	Open
	WHO_L: WHO Lambda index used to compute the WHO z-score WHO_M: WHO Mu index used to compute the WHO z-score WHO_S: WHO Sigma index used to compute the WHO z-score WHO_zScore: WHO z-score WHO_percentile: WHO percentile based on WHO z-score	Numeric data	Restricted
	WHO Weight status: WHO weight status based on WHO percentile	Category data: Underweight; Normal; Overweight; Obese	Open
	CDC_L: CDC Lambda index used to compute the CDC z-score CDC_M: CDC Mu index used to compute the CDC z-score CDC_S: CDC Sigma index used to compute the CDC z-score CDC_zScore: CDC z-score CDC_percentile: CDC percentile based on CDC z-score	Numeric data	Restricted
	CDC Weight status: CDC weight status based on CDC percentile	Category data: Underweight; Normal; Overweight; Obese	Open
Community affiliation	Cultural community declared from the question: Which community do you feel you belong to?	Category data: Kanak or Melanesian; European Caledonian (born in New Caledonia); European, born in France or elsewhere; Wallisian, Futunian, Tahitian (Polynesian); Indonesian, Vietnamese or Asian of other origin; African; Other	Restricted
Multigroup ethnic identity measure	Six questions to measure the multigroup ethnic identity [7]	Category data: Not agree at all; Rather disagree; Somewhat agree; Completely agree	Open
Food frequency questionnaire	Thirty-three questions to get information about the participant food habits [8]	Category data in most of cases: from 5 to 7 choices. Numeric for one question: integer from 0 to 10. Text for 3 open questions.	Open
Self-esteem questionnaire	Ten questions about self-esteem [9,10]	Category data: Not agree at all; Rather disagree; Somewhat agree; Completely agree	Open
Procrastination assessment scale-student	Eleven questions to assess academic procrastination [11]	Category data: Not at all true for me; Rather not true for me; Neutral; Somewhat true for me; Completely true for me	Open
Sleep	Eight questions about sleep behavior	Category data: from 4 to 16 choices	Open
Multimedia devices	Five questions about multimedia devices owned	Category data: binary, 4 or 5 choices	Open
Screens and me	Six questions designed to understand the participants’ behavior regarding the use of screens	Category data: from 4 to 7 choices	Open
Screens and information	Twenty questions to understand how the participants use the multimedia devices to get information, especially regarding food	Category data: binary, from 4 to 7 choices Text for 3 open questions	Open
My body and me	Twenty-one questions to assess the participants’ satisfaction regarding their own body [12]	Category data: Strongly disagree; Very little agree; Little agree; Somewhat agree; Agree a lot; Strongly agree	Open
My appearance, my family, my friends and the media (Sociocultural Attitudes Towards Appearance Questionnaire-4: SATAQ-4)	Twenty-two questions to assess the participants’ opinion regarding their physical appearance as well as the pressure the participants felt about their physical appearance from family, peers and media [13,14]	Category data: Completely disagree; Somewhat disagree; Neither agree nor disagree; Somewhat agree; Completely agree	Open
My body image	Three questions to assess the participants’ body image and their ideal weight; the image shown corresponded to the participant's declared gender [15]	Ordinal category data: integer from 1 to 27	Open
Aerobic fitness	Maximal aerobic speed (MAS) test in km/h	Numeric data: [7,17]	Open
	Maximal oxygen consumption (VO_2_max) assessment in mL/min/kg	Numeric data: [26.6, 78.2]	Restricted

⁎ Because of the calculation process consisting in rounding off the age in months divided by 12, the resulting age in years of the oldest participant is 17 (i.e., the 199/12 approximation).

This is a dataset with 1062 participants. However, since information was collected on different days (c.f. EXPERIMENTAL DESIGN, MATERIALS AND METHODS section), the number of participants can be different from a section to another. That is why the dataset contains complete formation regarding:

• anthropometric measurements, impedancemeter measurements, and weight status sections for 1011 participants
• community affiliation section for 1022 participants
• multigroup ethnic identity measure, food frequency questionnaire, self-esteem questionnaire, procrastination assessments scale-student, and sleep sections for 1031 participants
• multimedia devices, screens and me, screens and information, and my body and me sections for 1010 participants
• fitness section for 334 participants

### 3.2 Description of the participants

Table 3 describes the participants. There are missing data regarding sex for one participant, ethnic community for 40 participants and SES for 9 of them. About 49 % were females and 36 % lived in a rural environment. Around a third were under the age of 12 and around a half were aged between 13 and 14. Around a third of the participants declared to be Europeans, 44 % Melanesians and 10 % Polynesians. Around 58 % of the participants were from Low SES and 14 % from Medium SES.

**Table 3:** Description of the participants.

Factor	Category	N ( %)
Sex	Female	521 (49.1)
	Male	540 (50.8)
Place of living	Rural	384 (36.2)
	Urban	678 (63.8)
Age range	Less than or equal to 12	378 (35.6)
	Between 13 and 14 inclusive	500 (47.1)
	Greater than or equal to 15	184 (17.3)
Ethnic community*	European	386 (36.3)
	Melanesian	468 (44.1)
	Polynesian	103 (9.7)
	Other	65 (6.1)
SES	Low	616 (58.0)
	Medium	144 (13.6)
	High	293 (27.6)

*Primary categories found in the restricted dataset have been merged here as follows: "European Caledonian (born in New Caledonia)", "European, born in France or elsewhere" into “European”; "African", "Indonesian, Vietnamese or Asian of other origin" and "Other" into “Other”.

### 3.3 Distribution of anthropometric measurements and weight status

Table 4 describes the main anthropometric measurements.

**Table 4:** Description of the anthropometric measurements.

Instrument	Measure (unit)	Minimum	Average	Maximum
Tanita HR 001	Height (m)	1.30	1.58	1.93
Tanita DC-430MA	Weight (kg)	25.0	53.5	131.6
	BMI (kg/m²)	13.5	21.1	49.5
Holtain skinfold callipers	Bicipital (mm)	2.4	8.3	89.0
	Tricipital (mm)	3.8	13.4	54.4
	Suprailiac (mm)	3.7	17.1	114.3
	Subscapular (mm)	3.2	17.4	88.8

Four standards used to determine the participant weight status. The column name is defined as follows: <standard><weight status component>. The standards considered were: International Obesity Task Force (IOTF) [16], Cachera [17], World Health Organization (WHO) [18] and Center for Disease Control and Prevention (CDC) [19,20]. The weight status components are weight status, z-score, percentile and information needed to compute z-score (i.e., lambda (L), mu (M) and sigma (S)).

Table 5 describes the main derivations of the anthropometric measurements, i.e. z-scores and weight status for each standard considered in the dataset. According to the standard, between a quarter and a third of participants were considered overweight or obese.

**Table 5:** Basic statistic description of z-scores and weight status.

Standard	Minimum	Average	Maximum
IOTF z-score	−3.01	0.65	3.79
Cachera z-score	−2.99	0.96	5.44
WHO z-score	−3.36	0.56	4.87
CDC z-score	−3.50	0.41	2.72

Standard	Category		N ( %)

IOTF	Underweight		76 (7.2)
	Normal		625 (58.9)
	Overweight		206 (19.4)
	Obese		104 (9.8)
Cachera	Underweight		17 (1.6)
	Normal		729 (68.6)
	Overweight		265 (25.0)
WHO	Underweight		18 (1.7)
	Normal		628 (59.1)
	Overweight		219 (20.6)
	Obese		146 (13.7)
CDC	Underweight		32 (3.0)
	Normal		664 (62.5)
	Overweight		177 (16.7)
	Obese		138 (13.0)

**Fig. 1:**
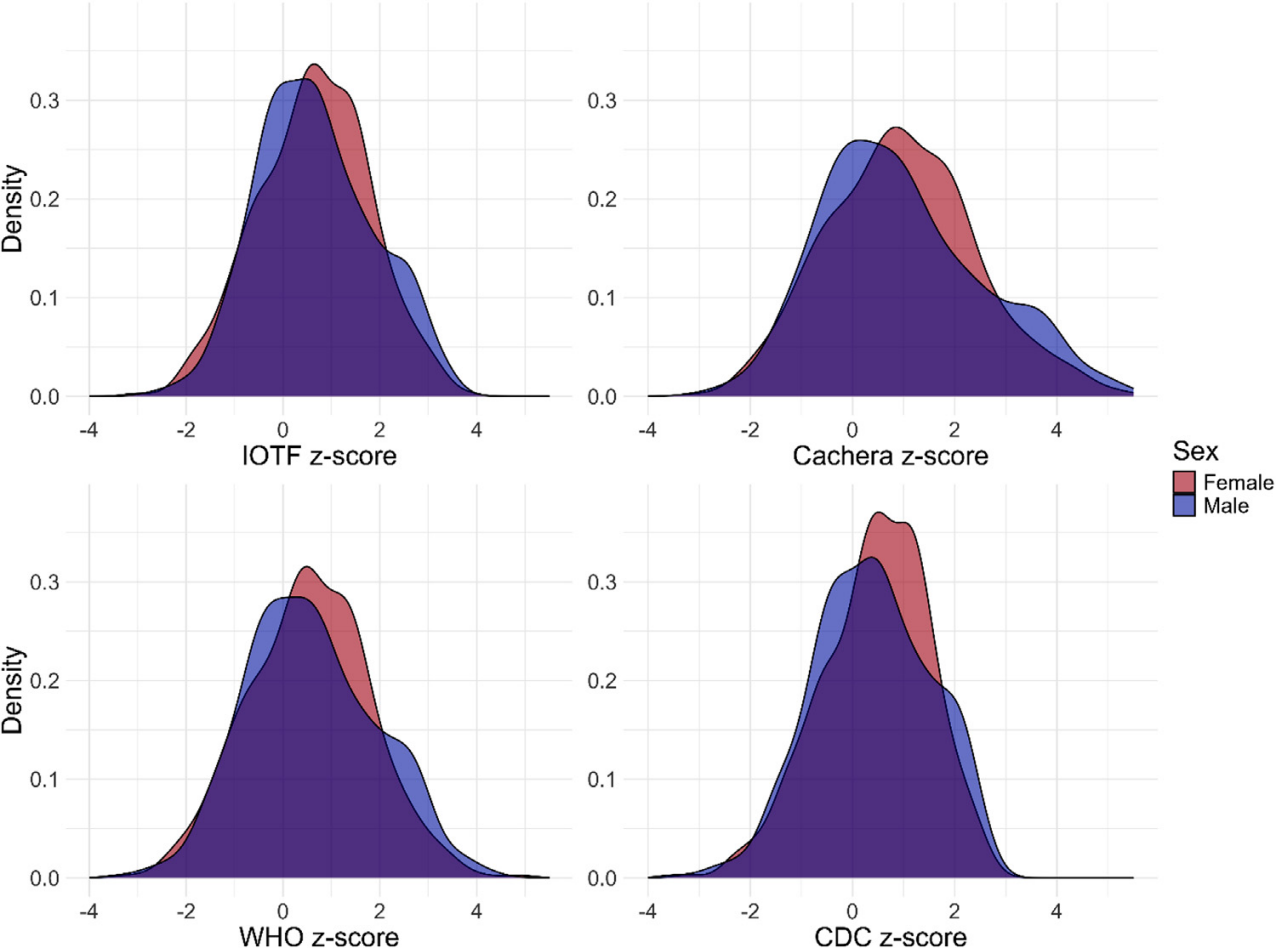
Distribution of the z-scores related to four standards: (a) IOTF, (b) Cachera, (c) WHO, (d) CDC.

Fig. 1 completes the anthropometric data by showing the z-score distributions.

### 3.4 Questionnaires

Various figures displaying the frequency of the participants’ responses for each questionnaire section are proposed in supplementary materials. Readers can consider “Food cultures_dataContentDescription_en.pdf” available in the anonymized Zenodo deposit [5].

### 3.5 Aerobic fitness

**Fig. 2:**
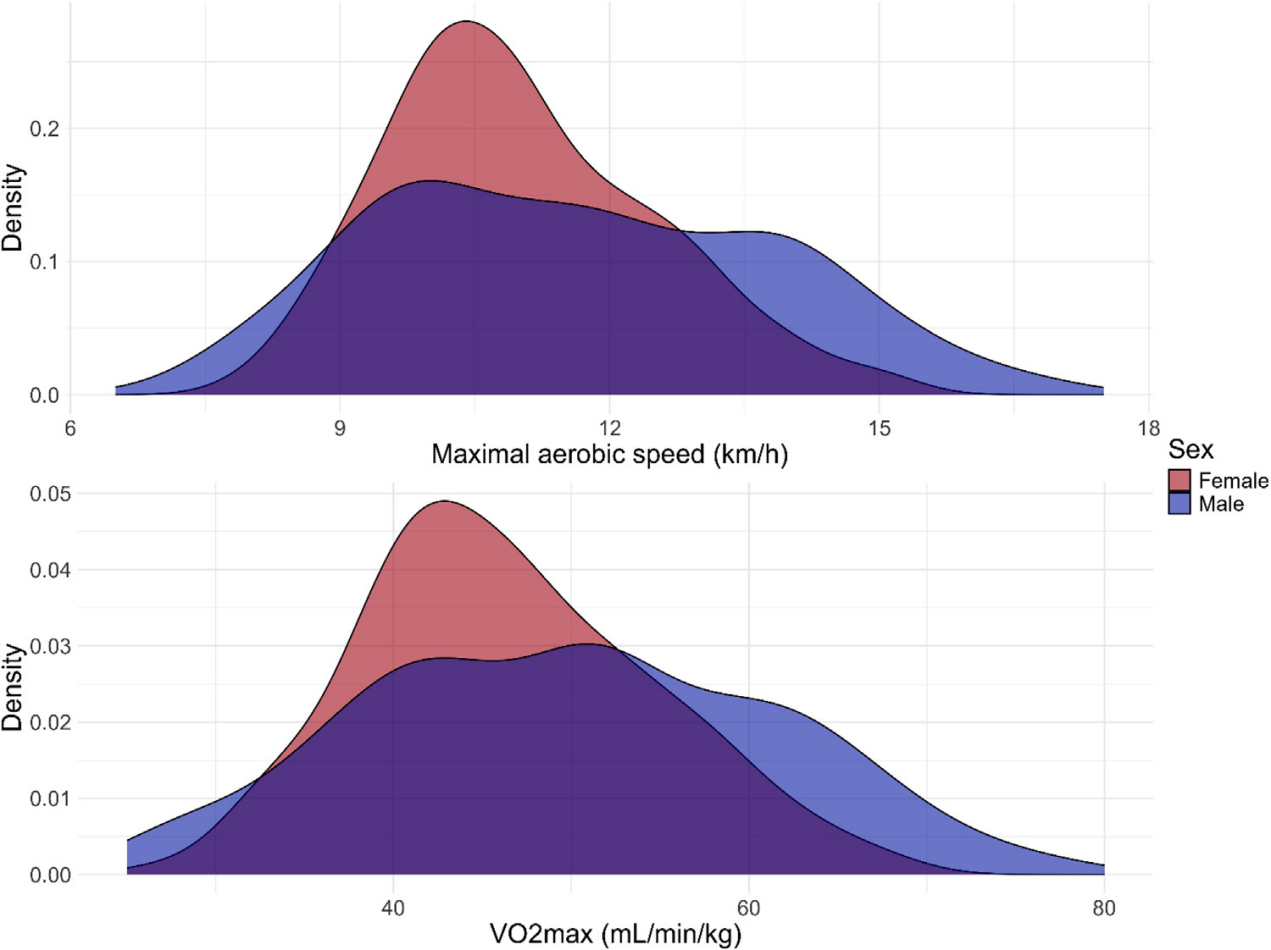
Distributions according to sex of (a) maximal aerobic speed and (b) assessed VO_2_max.

Table 6 describes the frequencies and Figure 11 shows the distributions of MAS values and VO_2_max assessed from the MAS test [21]. 334 participants took part in the fitness test (Fig. 2).

**Table 6:** Frequencies and percentages of fitness measures: Maximal aerobic speed and VO_2_max.

Fitness measure	Category	N ( %)
Maximal aerobic speed - MAS (km/h)	MAS≤11	178 (53.3)
	11<MAS≤14	130 (38.9)
	14<MAS≤16	23 (6.9)
	16<MAS	3 (0.9)
VO_2_max (mL/min/kg)	$VO_{2}\max\leq 45$	143 (42.8)
	$45<VO_{2}\max\leq 58$	126 (37.7)
	$58<VO_{2}\max\leq 65$	44 (13.2)
	$65<VO_{2}\max\leq 68$	10 (3.0)
	$68<VO_{2}\max\leq 70$	5 (1.5)
	$70<VO_{2}\text{max}$	6 (1.8)

## 4 Experimental Design, Materials and Methods

### 4.1 Context

New Caledonia is a South Pacific archipelago located between 162–169° E longitude and 19–23° S latitude. It is divided in three administrative provinces (Loyalty Islands Province, Northern Province and Southern Province) with differences in terms of urbanization and ethnic composition. In 2019, there were 271,407 inhabitants: 75 % lived in Southern Province, 18 % lived in Northern Province and 7 % in Loyalty Islands Province. Around 67 % of the total population lived in Grand Noumea which is the only urban area in the archipelago [22]. Among the 33 secondary public schools, 40 % were located in rural areas and 60 % in urban areas [23].

### 4.2 Recruitment of participants

For data collection, 8 secondary public schools were randomly selected according to (1) a school size criterion (*n* > 200) to ensure sufficient data in a single field trip (2) and a representative repartition between rural and urban areas. For each selected school, the project was introduced to the school principal who gave her/his approval. In the French school system, a secondary school is organized in four class level, from 6th to 3rd which correspond to Year 7 to Year 10 in the English school system. One or two classes per class level were randomly selected by a school staff member in order to get approximately 150 participants in 6 different classes. Field trips were organized between July 2018 and April 2019.

### 4.3 Experimental conditions

Data was collected over two consecutive days during class time. At least two members of the research team were in the classroom for each phase of data collection, especially to clarify or answer any questions about the questionnaires. The anthropometric measurements were taken by or with the school nurse and recorded by a trained staff of the research team.

The MAS fitness test took place during physical education classes. Performance was measured and recorded by the teacher.

### 4.4 Personal information

Place of living (rural or urban) was determined according to the school location and the criterion used by the European standard to assess the degree of urbanization [24]. The participants who attended one of the three rural schools were classified as “Rural” and the others were classified as “Urban”.

Other information collected from the school establishments included sex, birthdate and the professional activity of the participants’ legal representatives. From the birth date and the date of the data collection, the age in months could be deduced. Then, age in years and age range (“Less than or equal to 12”, “Between 13 and 14 inclusive”, “Greater than or equal to 15”) were trivially calculated.

From the professional activity of the participants’ legal representatives, four socioeconomic categories were determined [25]. Then, the socioeconomic status (SES) was indexed on the basis of the occupation of the household reference person (defined as the householder with the highest income) using the National Statistics Socio-Economic classification [26]. From this basis, three categories were generated and noted as the International SES: managerial and professional occupations (High), intermediate occupations (Medium), and routine and manual occupations (Low).

### 4.5 Anthropometric measurements

The anthropometric measurements were taken by or with the school nurse at the school sick bay while the participants were in light clothing. Height, weight, waist circumference and skinfolds were measured. Height was measured to the nearest 0.1 cm with a portable stadiometer: Leicester Tanita HR 001 (Tanita Corporation, Tokyo, Japan). Weight was measured to the 0.1 kg with an impedance scale: Tanita DC-430MA (Tanita Corporation, Tokyo, Japan). Waist circumference was measured at the midpoint between the lowest rib and the iliac crest. The measuring tape was placed horizontally, and measurements were done twice to the nearest 0.1 cm at the end of a normal expiration. When the difference between the two records was greater than 0.5 cm, a third measurement was taken, and the average of the nearest values was kept and recorded. The thickness of triceps, biceps, supra-iliac and subscapular skinfolds was measured twice to the nearest 0.1 mm with Holtain skinfold callipers.

### 4.6 Weight status

Body mass index (BMI) was deduced from weight (in kg) and weight (in m) measurements. Weight status classifications were computed according to four BMI-based definitions: IOTF references [16], French references [17], WHO references [18], and CDC references [19,20]. For each definition, reference tables contain reference centiles and LMS reference values used to determine weight status classification and to compute the BMI z-score (denoted by z in the following) [27].

Regarding the IOTF reference, the weight status is determined from sex and age in months. A participant whose BMI is:

• less than the C.18.5 value is considered as underweight
• between the C.18.5 and C.25 values is considered as normal
• between the C.25 and C.30 values is considered as overweight
• greater than the C.30 value is considered as obese

Regarding the Cachera reference, the weight status is determined from sex and age in decimal years. A participant whose BMI is:

• less than the 3e value is considered as underweight
• between the 3e and 97e values is considered as normal
• greater than the 97e value is considered as overweight

Regarding the WHO reference, the weight status is determined from sex and age in months [28]. A participant whose BMI is:

• less than the SD2neg (−2 SD) value is considered as underweight
• between the SD2neg (−2 SD) and SD1 (1 SD) values is considered as normal
• between the SD1 (1 SD) and SD2 (2 SD) values is considered as overweight
• greater than the SD2 (2 SD) value is considered as obese

The WHO z-score was also corrected as recommended when |z|>3. Let’s note $z^{*}$ the corrected value, then it was computed as follows:

$$z^{*}=\left\{ \begin{array}{cc} z & \mathrm{if}\;\left|z\right|\leq 3\\ 3+\left(\frac{\text{BMI}-\text{SD}3}{\text{SD}3-\text{SD}2}\right) & \mathrm{if}\;z>3\\ -3+\left(\frac{\text{BMI}-\text{SD}3\text{neg}}{\text{SD}2\text{neg}-\text{SD}3\text{neg}}\right) & \mathrm{if}\;z<-3 \end{array}\right.$$

Where SD2, SD3, SD2neg and SD3neg are provided in the WHO reference tables. These values are also computable as follows:

$$\left\{ \begin{array}{c} \begin{array}{c} \text{SD}2=M\;\times {\left(1+L\times S\times 2\right)}^{1/L}\\ \text{SD}3=M\;\times {\left(1+L\times S\times 3\right)}^{1/L} \end{array}\\ \begin{array}{c} \text{SD}2\text{neg}=\;M\;\times {\left(1-L\times S\times 2\right)}^{1/L}\\ \text{SD}3\text{neg}=M\;\times {\left(1-L\times S\times 3\right)}^{1/L} \end{array} \end{array}\right.$$

Regarding the CDC reference, the weight status is determined from sex and age in months. A participant whose BMI is:

• less than the percentile 5 (P5) value is considered as underweight
• between the percentile 5 (P5) and percentile 85 (P85) values is considered as normal
• between the percentile 85 (P85) and percentile 95 (P95) values is considered as overweight
• greater than the percentile 95 (P95) value is considered as obese

### 4.7 Body composition

From the measurements of skinfolds, we computed the sum of skinfolds (Σ) in mm. This value enabled to assess the density, and then deduce fat body mass (FBM) in percentages and lean body mass (LBM) in kg [29]. Here are the formulas we used for these assessments:

$$\text{Density}=\;\left\{ \begin{array}{c} 1.1533-0.0643\;\times \log\left(\Sigma \right)\;\text{for}\;\text{males}\\ 1.1369-0.0598\;\times \log\left(\Sigma \right)\;\text{for}\;\text{females} \end{array}\right.$$

$$\text{FBM}=\;\left(\frac{4.95}{\text{Density}}-4.5\right)\times \;100\;\text{and}\;\text{LBM}=\text{Weight}\;\times \left(1-\frac{\text{FBM}}{100}\right).$$

### 4.8 Completion of questionnaires

The questionnaires completed by the participants were divided in 11 sections, 5 of which were completed on the first day in the field (first part) and the other 6 on the second day (second part), which should correspond to a completion time of about 45–50 min per session.

The first part of the questionnaires starts with the “Community affiliation” and an abbreviated version of the “Multigroup ethnic identity measure” sections whose primary version was used in prior research by [7]. The “Community affiliation” is a 7-option question about the cultural community of the participants who can choose one of the most common cultural community found in New Caledonia or the “Other” option. The abbreviated version of the “Multigroup ethnic identity measure” consists of 6 items that use 4-option Likert scale: 1) Not agree at all; 2) Rather disagree; 3) Somewhat agree; 4) Completely agree. The average calculation of the 6 items provides an assessment of the cultural affiliation with higher scores reflecting greater affiliation. An example of application can be found in [30].

“Food frequency questionnaire” (FFQ) section is a slight adaptation to New Caledonia context from the prior Gwynn and colleagues’ research [8]. The main purpose of this section is to get information about participants’ behavior regarding their quantity and quality food consumption. It contains 33 items, 26 of which are 5-to-7-option closed questions regarding foods and beverages that participants may usually consume. A 11-option question (from 0 to 10) aims to assess the quantity of sugar participants may add in their drinks and foods per day. A binary question (i.e., Yes/No) followed by an open choice question is asked to make sure that the current FFQ does not leave out foods that are likely to be eaten. Finally, two 6-option questions and 2 open questions aims to know whether participants use to buy food products on the way from or to school, the purchase frequency and the type of products. Most of the responses can be converted to assess a daily consumption in participants. Examples of studies that used this FFQ can be found in [1,31,32].

“Self-esteem questionnaire” section is a French translation of the Rosenberg’s Self-Esteem scale validated by Vallieres and Vallerand [9,10]. It consists in 10 items whose answers are 4-option Likert scale: 1) Not agree at all; 2) Rather disagree; 3) Somewhat agree; 4) Completely agree. The items that are negatively formulated (3, 5, 8, 9, and 10) need to be reverse-scored for the total score calculation (i.e., 1 becomes 4, 2 becomes 3, and so on) so that the total score ranges from 10 to 40. Thus, self-esteem total score is simply calculated by adding the scores for each of the responses. This section has been used for instance in [33].

“Procrastination assessment scale – students” section is based on the Academic procrastination scale translated by [11] containing 11 items whose answers are 5-option Likert scale: 1) Not at all true for me; 2) Rather not true for me; 3) Neutral; 4) Somewhat true for me; 5) Completely true for me. Two items (4 and 6) need to be reverse-scored to calculate the score mean that ranges from 1 (low tendency to procrastinate) to 5 (high tendency to procrastinate) and properly determine the participants’ procrastination degree. This section has been used in [30].

The first part of the questionnaire ends with “Sleep” section. It has been developed for this data collection but some items (i.e., wake-up time and onset sleep time) have only been adapted to school context from tools commonly used for sleep research studies, such as Pittsburgh Sleep Quality Index. It contains 8 items that are closed questions whose purpose is to assess: 1) sleep duration on both weekdays and weekends; 2) the lack of sleep for participants; 3) potential causes of a delay in onset sleep time by using devices on evening for instance. Some items of this section have already been used in studies like in [1] and [32].

The second part of the questionnaires starts with “Multimedia devices” section that contains 5 items and was developed for this data collection. Three items are binary questions (i.e., Yes/No) and the others are 5-option-responses with single response. The purpose of this section is to assess the participants’ access and ownership regarding multimedia devices like television, computer, tablet and mobile phone. An example of output and application can be found in [31].

“Screens and me” section contains 6 items that are closed questions with single response developed for this data collection. The purpose of this section is to assess the time spent by participants in front of their screen during week days and weekends. Several items of this section were also used in [1,31].

“Screens and information” section contains 20 items developed for this data collection. Six items are closed questions with multiple responses, 8 items are binary questions (i.e., Yes/No), 3 items are closed questions with single response, and 3 items are open choice questions aiming to get deeper information about a previous close or binary question. The main purpose of this section is to understand the type of actions in which participants are engaged with their digital devices and how they collect information, especially regarding food.

“My body and me” section contains 21 items from the Physical Self-Inventory (PSI) that have been used in prior research by [12] and whose answers are based on a 6-option Likert scale: 1) Strongly disagree; 2) Very little agree; 3) Little agree; 4) Somewhat agree; 5) Agree a lot; 6) Strongly agree. The items describe global self-concept, physical self-worth, physical condition, sport competence, physical attractiveness, and physical strength. A selection of 18 items can be used for the short form and 12 items for the very short form of PSI.

“My appearance, my family, my friends and the media” section contains 22 items from the Sociocultural Attitudes Towards Appearance Questionnaire 4 (SATAQ-4) that have been used in prior research [13,14]. SATAQ-4 can be used “to assess internalization of appearance ideals and appearance related pressures” [14], relatively to 5 factors: 1) Internalization – Thin/Low Body Fat (5 items); 2) Internalization – Muscular/Athletic (5 items); 3) Pressures – Family (4 items); 4) Pressures – Peers (4 items); 5) Pressures – Media (4 items). The answers are 5-option Likert-scale with higher scores indicating greater internalization and endorsement of societal appearance ideals: 1) Completely disagree; 2) Somewhat agree; 3) Neither agree nor disagree; 4) Somewhat agree; 5) Completely agree.

The second part of the questionnaire ends with “My image” section that contains 3 items from a BMI-based Silhouette Matching Test (BMI-SMT) whose purpose is to assess the perceptions of ideal and current body images. BMI-SMT has been used in prior research [15]. The first item consists in a binary question by asking the participants if they are boys or girls so that the displayed silhouette image matches with their choice. Then, participants choose a reference number on a scale with 27 discrete choices on a scale from 1 to 27 representing BMI increasing relatively to 1) their current appearance, and 2) their ideal appearance. This section has also been used for instance to measure body satisfaction of participants in [33].

**Aerobic fitness**

Aerobic fitness was assessed with the MAS test described in [21]. A soundtrack imposed the rhythm around an indoor track with plastic cones placed at 20-m intervals. A participant aimed to reach each cone at each beep. The soundtrack was recorded so that the participant increased the running speed by 1 km/h per minute. The last stage reached by the participant was converted into MAS following the Léger et al. 1988′s formula [21,34].

We then determined an assessment of VO_2_max following this formula:

$$VO_{2}\max=31.025+3.238\;\times \;\text{MAS}-3.248\;\times \;\text{Age}+0.1536\;\text{MAS}\;\times \;\text{Age}$$

Where Age is the participant’s age in years and MAS the previous value determined with the MAS fitness test [21].

## Limitations

This dataset contains information on 1062 adolescent participants but, as data sources were diverse, there are missing data showed in Table 6.

During the completion of the question related to the cultural community (i.e., “Which community do you feel you belong to?”) during the first questionnaire session, there was a technical issue that the research team was unable to explain, resulting in the loss of most of the information provided just for this specific question. That is why this question was put again to the participants during the second questionnaire session, explaining why this dataset contains this information for 1022 participants. As a consequence, 193 participants answered twice. For those (*n* = 15) who provided inconsistent answers, i.e. providing different cultural communities, their answers were de-anonymized to keep the most probable answer regarding their personal information (Table 7).

**Table 7:** Missing data in the dataset.

Information	Number of missing data
Sex	1
Socioeconomic status	9
Anthropometric measurements	51
Tanner status	60
Impedancemeter measurements	51
First questionnaire session	31
Second questionnaire session	52

Moreover, inconsistent answers were recorded for the following question in the “Screens and me” section: “How much time do you spend in front of the computer or tablet during the weekend?”. The answer “Option 7” is recorded for 5 participants while this is a 6-choice question. We left this as it is in the current dataset.

## Ethics Statement

The research met all legal requirements mandated by the Declaration of Helsinki. The protocol was approved by the Ethics Committee of New Caledonia (CCE 2018–06 001). All parents received an information letter and gave written informed consent prior to the adolescents’ participation by means of a note to be signed in the correspondence booklet, and the adolescents gave written informed consent to their own participation.

## Credit Author Statement

Guillaume Wattelez: Conceptualization, Methodology, Software, Formal analysis, Investigation, Data curation, Writing – original draft, Writing – review & editing, Visualization. Stéphane Frayon: Methodology, Investigation. Akila Nedjar-Guerre: Investigation, Writing – review & editing. Corinne Caillaud: Investigation, Writing – review & editing. Olivier Galy: Methodology, Investigation, Writing – review & editing, Supervision, Funding acquisition.

## Data Availability

ZenodoAnthropometric measurements, questionnaire responses and fitness data collected during the scientific project: "Eating cultures and behaviors of young people in French-speaking Pacific countries in th (Original data)
ZenodoAnthropometric measurements, questionnaire responses and fitness data collected during the scientific project: "Eating cultures and behaviors of young people in French-speaking Pacific countries in th (Original data)
